# Cross-Border Transmissions of the Delta Substrain AY.29 During Tokyo Olympic and Paralympic Games

**DOI:** 10.3389/fmicb.2022.883849

**Published:** 2022-08-03

**Authors:** Takahiko Koyama, Reitaro Tokumasu, Kotoe Katayama, Ayumu Saito, Michiharu Kudo, Seiya Imoto

**Affiliations:** ^1^IBM Thomas J. Watson Research Center, Yorktown Heights, NY, United States; ^2^IBM Research–Tokyo, Tokyo, Japan; ^3^Laboratory of Sequence Analysis, Human Genome Center, The Institute of Medical Science, The University of Tokyo, Tokyo, Japan; ^4^Division of Health Medical Intelligence, Human Genome Center, The Institute of Medical Science, The University of Tokyo, Tokyo, Japan

**Keywords:** Olympic Games, Paralympic Games, SARS-CoV-2, COVID-19, delta variant, AY-29, cross-border transmission

## Abstract

Tokyo Olympic and Paralympic Games, postponed for the COVID-19 pandemic, were finally held in the summer of 2021. Just before the games, the Alpha variant was being replaced with the more contagious Delta variant. AY.4 substrain AY.29, which harbors two additional characteristic mutations of 5239C > T (NSP3 Y840Y) and 5514T > C (NSP3 V932A), emerged in Japan and became dominant in Tokyo by the time of the Olympic Games. Variants of SARS-CoV-2 genomes were performed to extract AY.29 Delta substrain samples with 5239C > T and 5514T > C. Phylogenetic analysis was performed to illustrate how AY.29 strains evolved and were introduced into countries abroad. Simultaneously, ancestral searches were performed for the overseas AY.29 samples to identify their origins in Japan using the maximum variant approach. As of January 10, 2022, 118 samples were identified in 20 countries. Phylogenetic analysis and ancestral searches identified 55 distinct introductions into those countries. The United States had 50 samples with 10 distinct introductions, and the United Kingdom had 13 distinct strains introduced in 18 samples. Other countries or regions with multiple introductions were Canada, Germany, South Korea, Hong Kong, Thailand, and the Philippines. Among the 20 countries, most European and North American countries have vaccination rates over 50% and sufficient genomic surveillances are conducted; transmissions seem contained. However, propagation to unvaccinated regions might have caused unfathomable damages. Since samples in those unvaccinated countries are also undersampled with a longer lead time for data sharing, it will take longer to grasp the whole picture. More rigorous departure screenings for the participants from the unvaccinated countries might have been necessary.

## Introduction

Long-awaited Tokyo 2020 Olympic and Paralympic Games were postponed for a year due to the COVID-19 pandemic. Despite the overwhelming opposing Japanese public opinions, parties, including International Olympic Committee (IOC), International Paralympic Committee (IPC), the Japanese Government, and Tokyo Metropolitan Government, decided to hold the events in the summer of 2021, starting July 23 and August 24, respectively without spectators in the venues. They made efforts to reduce the number of visitors outside of Japan to minimize the risk of importing exogenous novel SARS-CoV-2 strains; as a result, it was substantially reduced to 54,250 from the pre-pandemic estimate of 180,000 (NHK, 2021a; Yomiuri Shimbun, 2021).

**FIGURE 1:**
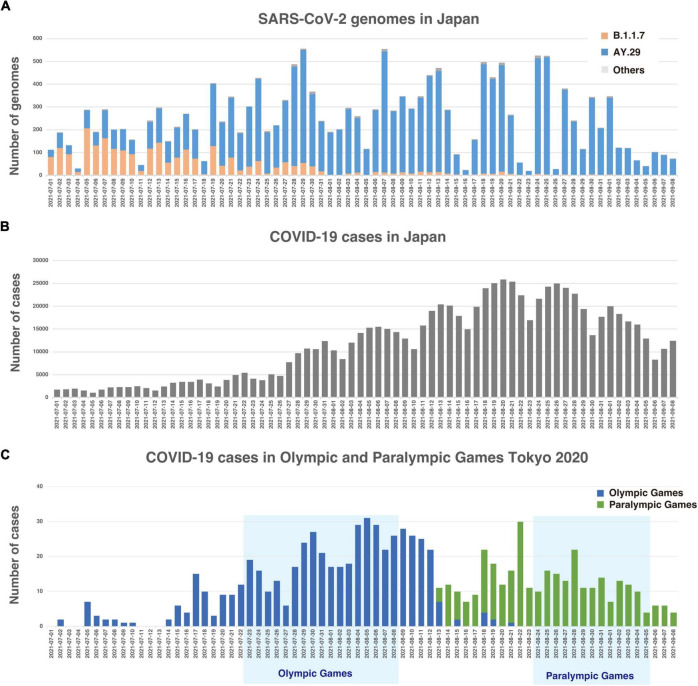
**(A)** Histogram of strains by lineages in Tokyo. **(B)** Number of daily new COVID-19 positive cases in Japan. **(C)** Number of daily new COVID-19 positive cases among Olympic and Paralympic participants.

Just before the Olympic Games began, in Japan, the Alpha variant [PANGO lineage (Rambaut et al., 2020): B.1.1.7] was being replaced by the Delta variant, which harbors T478K and L452R mutations in spike protein, with a surge of patients due to highly infectious nature of the Delta variant (Figure 1A; Michael Rajah et al., 2021). Japanese Government declared a state of emergency in many prefectures, including Tokyo and neighboring prefectures, to mitigate the risk of potential healthcare system collapse. A combination of reduction of mobilities, mask compliance, and an increase in vaccination rate appeared to have attributed to the significant reduction of positive cases by the end of September (Coronavirus Pandemic (COVID-19), 2020) as shown in Figure 1B. In fact, the reproduction number had already fallen below one in Tokyo by mid-August (National Institute of Infectious Disease, 2021), just 1 week after the end of the Olympic Games.

Despite the 72-h testing requirement, Japanese airport quarantine stations identified 54 positive cases at the border control among overseas participants (Supplementary Figure 1). During the period, a total of 863 positive cases was identified (Figure 1C; COVID-19 Positive Case List, 2021). Out of 863 positive cases, 174 cases belong to the Olympic overseas visitors and 80 cases belong to Paralympic overseas visitors (NHK, 2021b). Therefore, the majority of the positive cases belong to Japanese residents, such as contract workers and volunteers.

There have been two major concerns for the events regarding COVID-19 since athletes from 205 countries or regions compete in the events. First, Japanese citizens were afraid of novel exogenous strains will be introduced into the Japanese population by the participants from abroad, who are waived for any self-quarantine (Wells et al., 2021). Secondly, variants of concerns (VoC) and variants of interest (VoI) strains besides novel strains, are exported back with the returning participants to the unvaccinated regions. Vaccination rates in low-income countries are still below 5% (Coronavirus Pandemic (COVID-19), 2020) and introduction to highly infective strains can make devastating outcomes in these areas.

Impartial scientific evaluation of how mass gathering events, such as the size of the Olympic and Paralympic Games, affect cross-border transmissions must be conducted. In this study, we have analyzed the SARS-CoV-2 strains transmitted outside of Japan during the time of the Olympic and Paralympic Games.

## Materials and Methods

To perform our analysis, 6,783,483 full genomes extracted from human subjects, were downloaded from the Global Initiative on Sharing Avian Influenza Data (GISAID) (Elbe and Buckland-Merrett, 2017; Shu and McCauley, 2017) and the National Center for Biotechnology Information (NCBI) up to January 10, 2022. 4,378,170 met a data quality criterion of less than 200 bp gap in an entire genome and excluded low coverage to avoid artifacts due to sequencing errors. However, there are some AY.29 strain genomes that are misclassified to AY.4 and some AY.4 strains were misclassified to AY.29. To rectify misclassification issues, 5514T > C (NSP3 V932A) and synonymous mutation of 5239C > T was used as criteria and definition of AY.29 for further analysis.

**FIGURE 2:**
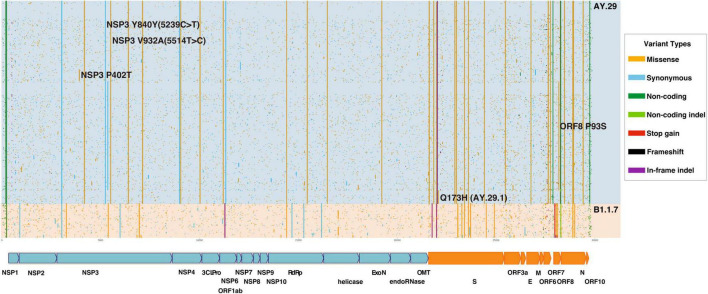
Variant Analysis of SARS-CoV-2 genomes sampled in Tokyo, Japan between July 1st and September 10, 2021. AY.29 Delta substrain is dominant in the period. Variants are colored depending on the type of mutations (missense, synonymous, non-coding, stop-gained, and frameshift). 3CLPro, 3C like protease; del, deletion; delins, deletion–insertion; E, envelope protein; ExoN, 3’-5’ exonuclease; M, membrane glycoprotein; N, nucleocapsid phosphoprotein; NA, not applicable; NSP, non-structural protein; OMT, *O*-methyltransferase; ORF, open reading frame; RdRp, RNA-dependent RNA polymerase; SARS-CoV-2, severe acute respiratory syndrome coronavirus 2; S, spike glycoprotein; UTR, untranslated region.

Variant annotation of SARS-CoV-2 genomes was performed as described in our previous report (Koyama et al., 2020). In a nutshell, the strain was first aligned in a pairwise manner with NC_045512 SARS-CoV-2 reference genome using the Needleman-Wunsh algorithm (Needleman and Wunsch, 1970) using a needle in EMBOSS version 6.6.0.0 with a gap penalty of 100 and extension penalty of 0.5. From the pairwise alignment, differences with the reference genome were extracted as genome changes and subsequently, annotated for the types of mutation and amino acid changes if any. For the samples collected in Tokyo between 1 July and 10 September, we draw a plot of variants as illustrated in Figure 2.

Phylogenetic analysis was carried out on all the AY.29 strains exported and randomly selected 1,000 AY.29 genomes from Japan. This selection consists of genomes with full collection date information and no unknown bases or gaps. We first aligned sequences using MAFFT version 7.475 (Katoh et al., 2002). Subsequently, we used Bayesian evolutionary analysis by sampling trees (BEAST), version 2.6.6. for 10,000,000 chain length employing the Hasegawa-Kishino-Yano mutation model (Hasegawa et al., 1985), with the strict clock mode and with a coalescent exponential population in the prior setting. For an overseas sample without exact date information, day 15 was assigned. For visualization, we used ggtree version 2.0.4 (Yu, 2020) on R version 3.6.3 (R Core Team, 2021) with country and subtype information.

Similarly, an ancestral strains search was performed for the overseas AY.29 samples using the maximum variant approach (Tokumasu et al., 2021). An ancestral strain should have a subset of mutations of the child strain. Among such ancestral strains, one with the maximum common variants is considered as an immediate ancestor or a parent in an ideal situation. Nevertheless, in many samples in the Delta variants, spikes G142D, T95I, and 156_158delinsG are missing because of sequencing artifacts (Sanderson and Barrett, 2021). To find proper ancestral strains, these mutations were overlooked for our ancestral searches in this study.

## Results

It is apparent that the Delta substrain AY.29, which harbors two characteristic mutations of 5239C > T (NSP3 Y840Y) and 5514T > C (NSP3 V932A) (Abe and Arita, 2021), was the dominant strain during the time of Olympic and Paralympic Games in Tokyo as shown in Figure 2. Figure 1A shows that the Alpha variant (B.1.1.7) was being replaced by AY.29 around the end of June and early July before the Game started on 23 July.

AY.29 evolved from AY.4 in Japan in April acquiring 5239C > T and 5514T > C mutations (EPI_ISL_2723567/EPI_ISL_2723568). The chronological order of emergences of 5239C > T and 5514T > C is not clear from the data. The ancestral AY.4 was an exogenous strain as seen in EPI_ISL_1927416, which is collected from a traveler from India at a Japanese airport quarantine in April; however, the actual introduction of the ancestral AY.4 strain might be earlier than April. In spike protein, besides D614G, L452R, T478K, P681R, and D950N, almost all AY.29 strains have T19R, T19I, G142D, and 156_158delinsG in N-terminal Domain (NTD). Among the substrains of AY.29, one with ORF8 P93S forms the largest group followed by one with spike Q173H, which is now classified as AY.29.1.

**FIGURE 3:**
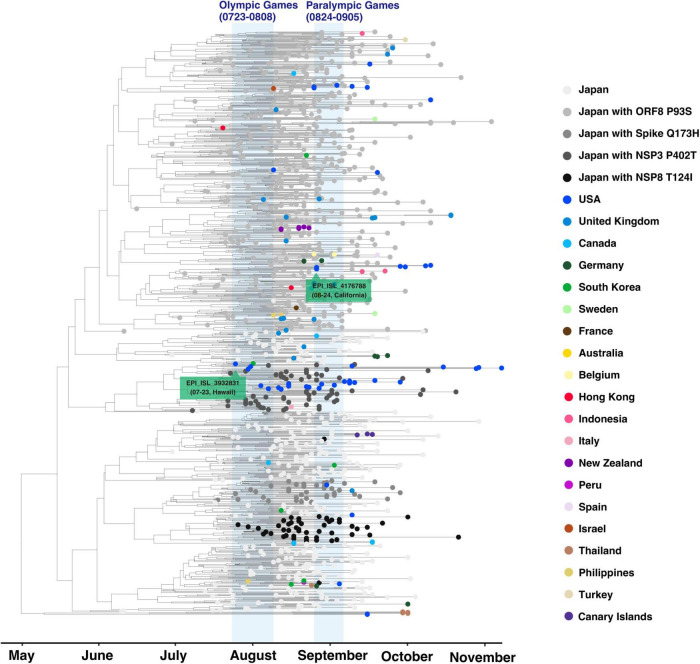
Phylogenetic analysis of AY.29 strains. All overseas samples were combined with randomly selected 1,000 AY.29 genomes in Japan. The exported strains of the two largest clusters in the AY.29 exported strains were labeled in green.

As of 10 January 2022, 118 of AY.29 exported samples were identified in 20 countries (Table 1). 50 samples in the United States were found, followed by 18 samples in the United Kingdom and eight samples in Canada. From phylogenic analysis as shown in Figure 3 and ancestral strain searches, 55 distinct AY.29 strains were known to be transmitted to the outside territories of Japan. The biggest overseas AY.29 cluster, which harbors NSP3 P402T mutation, occurred in Hawaii. The introduction of the strain took place before the Olympic Games; therefore, this Hawaiian cluster is not associated with the events. Another large cluster due to AY.29 strain with ORF8 P93S and NPS3 N873D mutations seems to be related to the United States Military stationed in Okinawa, the southern island prefecture; therefore, this strain is unrelated to the events as well. United Kingdom had 13 distinct AY.29 introductions, and the United States had 10 of them in the second place. Other countries or regions with multiple AY.29 introductions were Canada, Germany, South Korea, Hong Kong, Thailand, and the Philippines. The rest of the countries or regions, Italy, France, Spain, Sweden, Belgium, Peru, Australia, New Zealand, Israel, Indonesia, and Turkey, had a single AY.29 strain introduced. Furthermore, there were no incidents of indirect transmissions of AY.29 strains not involving Japan. Out of the exported 55 strains, Table 1 indicates that 41 of them were collected after August 1st and with their ancestral strains collected in Greater Tokyo Area, including Tokyo, Kanagawa, China, Saitama, and Ibaraki. In other words, exported AY.29 strains whose Japanese ancestral strains are found outside of Tokyo and its neighboring areas, such as Osaka, Hyogo, Gifu, Fukuoka, Kumamoto, and Okinawa, are unlikely related to the events. Furthermore, exported samples found in July are not likely related to the events regardless of the locations of their ancestral strains. There remains the possibility that these 41 strains are associated with the Olympic and Paralympic participants.

**TABLE 1:** AY.29 strains identified outside of Japan.

Country	AY29 strains with collection dates and locations	Characteristic variants	Japanese AY.29 ancestral strain
United States	EPI_ISL_3932831 (2021-07-23, Hawaii), EPI_ISL_3933098 (2021-07-28, Hawaii), EPI_ISL_3933071 (2021-07-29, Hawaii), EPI_ISL_3933081 (2021-08-02, Hawaii), EPI_ISL_3933062 (2021-08-05, Hawaii), EPI_ISL_3609214 (2021-08-09, California), EPI_ISL_4201347 (2021-08-10, Hawaii), EPI_ISL_4345621 (2021-08-13, Hawaii), EPI_ISL_4728273 (2021-08-13, Hawaii), EPI_ISL_4728362 (2021-08-13, Hawaii), EPI_ISL_4345610 (2021-08-20, Hawaii), EPI_ISL_4345585 (2021-08-21, Hawaii), EPI_ISL_4199055 (2021-08-23, Alaska), EPI_ISL_4728460 (2021-08-25, Hawaii), EPI_ISL_3943129 (2021-08-26, New York), EPI_ISL_4728404 (2021-08-31, Hawaii), EPI_ISL_5053721 (2021-09-06, Hawaii), EPI_ISL_5053784 (2021-09-08, Hawaii), EPI_ISL_5094157 (2021-09-26, Hawaii), EPI_ISL_5053755 (2021-09-04, Hawaii), EPI_ISL_5053763 (2021-09-06, Hawaii), EPI_ISL_6698696 (2021-09-07, Hawaii), EPI_ISL_7263984 (2021-09-16, Hawaii), EPI_ISL_6257008 (2021-10-13, North Dakota), EPI_ISL_5998608 (2021-10-26, North Dakota), EPI_ISL_5967340 (2021-10-27, North Dakota), EPI_ISL_6248505 (2021-11-05, North Carolina)	NSP3:P402T	EPI_ISL_3876536 (Tokyo)
	EPI_ISL_4176788 (2021-08-24, California), EPI_ISL_4176791 (2021-08-24, California), EPI_ISL_4176786 (2021-08-24, Mississippi), EPI_ISL_5084382 (2021-09-28, North Carolina), EPI_ISL_5230354 (2021-10-06, North Carolina), EPI_ISL_5230408 (2021-10-08, North Carolina), EPI_ISL_5238665 (2021-10-08, North Carolina), EPI_ISL_5084355 (2021-09-26, North Carolina)	ORF8:P93S, NSP3:N873D	EPI_ISL_4712063 (Okinawa)
	EPI_ISL_4176784 (2021-08-24, New Hampshire)	ORF3:S272G, NSP3:N873D	EPI_ISL_4888720 (Okinawa/US base)
	EPI_ISL_3749469 (2021-08-07, Ohio), EPI_ISL_3905086 (2021-08-23, Utah), EPI_ISL_3905136 (2021-08-23, Utah), EPI_ISL_3905142 (2021-08-23, Utah), EPI_ISL_3905301 (2021-08-23, Utah)	ORF8:P93S	EPI_ISL_2723565 (Tokyo)
	EPI_ISL_4761230 (2021-09-17, New York), EPI_ISL_4514414 (2021-09-14, New York)	ORF8:P93S, NSP2:T388I	EPI_ISL_2723565 (Tokyo)
	EPI_ISL_4910598 (2021-09-07, Missouri), EPI_ISL_4997950 (2021-09-13, Missouri), EPI_ISL_4545516 (2021-08-31, Minnesota)	ORF8:P93S, NSP16:T151I	EPI_ISL_3870758 (Ibaraki)
	EPI_ISL_4183744 (2021-09-01, California)	ORF8:P93S, NSP2:S36N, Spike:G1099D	EPI_ISL_4759537 (Tokyo)
	EPI_ISL_4914318 (2021-09-02, Wisconsin)	Spike:H146Q	EPI_ISL_2723567 (Tokyo)
	EPI_ISL_4182356 (2021-08-28, California)	NSP12:T76I, NSP1:M85del, NSP3:A1537S, Spike:Q173H	EPI_ISL_4708043 (Saitama)
	EPI_ISL_4812557 (2021-09-07, Illinois)	NSP3:I1413F, NSP3:N1284S, NSP3:V61I	EPI_ISL_3898844 (Tokyo)
United Kingdom	EPI_ISL_3573583 (2021-08-03, England), EPI_ISL_3883821 (2021-08-25, England)	ORF8:P93S, NSP13:I334V	EPI_ISL_2723565 (Tokyo)
	EPI_ISL_3437719 (2021-08-10, England), EPI_ISL_3574088 (2021-08-11, England)	ORF8:P93S, NSP3:Y1185C, NSP5:A260V	EPI_ISL_4702802 (Saitama)
	EPI_ISL_4531378 (2021-09-15, England), EPI_ISL_4531523 (2021-09-16, England)	ORF8:P93S, ORF7a:H73Y, NSP2:K67N	EPI_ISL_2723565 (Tokyo)
	EPI_ISL_3775708 (2021-08-15, England)	ORF3:D22Y, NSP12:L638F, NSP13:Y541C	EPI_ISL_4698359 (Tokyo)
	EPI_ISL_4530565 (2021-09-23, England)	ORF8:P93S, NSP1:R24C, NSP3:N444S, N:D402Y, N:R41Q, Spike:L5F	EPI_ISL_3896031 (Tokyo)
	EPI_ISL_4530448 (2021-09-21, England)	ORF8:P93S, NSP14:A353T, N:T24N	EPI_ISL_4757664 (Shizuoka)
	EPI_ISL_3471119 (2021-08-08, Scotland)	ORF8:P93S, NSP14:T372I, NSP2:A26V	EPI_ISL_3897760 (Japan)
	EPI_ISL_3574055 (2021-08-12, England), EPI_ISL_5484052 (2021-10-16), EPI_ISL_5484738 (2021-10-16)	ORF8:P93S, NSP2:K67N	EPI_ISL_2723565 (Tokyo)
	EPI_ISL_3528603 (2021-08-12, England)	ORF8:P93S, NSP6:Q208H	EPI_ISL_4696458 (Tokyo)
	EPI_ISL_4122814 (2021-09-07, England)	Spike:Q173H, Spike:S929I	EPI_ISL_3898182 (Tokyo)
	EPI_ISL_3775517 (2021-08-19, England)		EPI_ISL_2723567 (Tokyo)
	EPI_ISL_3471038 (2021-08-09, Scotland)		EPI_ISL_3882891 (Chiba)
	EPI_ISL_3574052 (2021-08-12, England)	ORF8:P93S, ORF3:W128L, NSP3:G250V	EPI_ISL_2723565 (Tokyo)
Canada	EPI_ISL_4409271 (2021-08, Ontario), EPI_ISL_4409465 (2021-08, Ontario), EPI_ISL_4409681 (2021-08, Ontario), EPI_ISL_4409854 (2021-08, Ontario)	NSP12:P227L, NSP3:S1682F, NSP3:T182I, N:S37P	EPI_ISL_3882454 (Tokyo)
	EPI_ISL_4221013 (2021-08-05, Quebec)	ORF8:A65V	EPI_ISL_3799899 (Kanagawa)
	EPI_ISL_4432932 (2021-08-22, British Columbia)	ORF8:P93S, NSP14:D496Y, NSP2:T439A	EPI_ISL_4718461 (Osaka)
	EPI_ISL_4001827 (2021-08, Ontario)	ORF8:P93S	EPI_ISL_2723565 (Tokyo)
	EPI_ISL_4433144 (2021-08-24, British Columbia)		EPI_ISL_3896646 (Tokyo)
Germany	EPI_ISL_4610540 (2021-09-16, North Rhine-Westphalia), EPI_ISL_4610164 (2021-09-17, North Rhine-Westphalia), EPI_ISL_4616094 (2021-09-21, North Rhine-Westphalia), EPI_ISL_3886279 (2021-09-24, North Rhine-Westphalia)	NSP12:L638F	EPI_ISL_4698359 (Tokyo)
	EPI_ISL_5122077 (2021-09-21, North Rhine-Westphalia)	ORF8:S67F	EPI_ISL_4692148 (Chiba)
	EPI_ISL_3878033 (2021-08-19, Berlin), EPI_ISL_4042783 (2021-08-26, Lower Saxony)	ORF8:P93S, NSP13:D204E, Spike:R408I, Spike:V622F	EPI_ISL_4719577 (Fukuoka)
South Korea	EPI_ISL_5924898 (2021-07-30)	NSP3:P402T	EPI_ISL_3876536 (Tokyo)
	EPI_ISL_5924900 (2021-08-10)	ORF3:T151I	EPI_ISL_4725150 (Okinawa)
	EPI_ISL_3869693 (2021-08-14)	NSP15:T105I	EPI_ISL_2768526 (Kanagawa)
	EPI_ISL_3869916 (2021-08-19)		EPI_ISL_2723567 (Tokyo)
	EPI_ISL_3869923 (2021-08-20)	ORF8:P93S, ORF3:D155Y, NSP13:A598V	EPI_ISL_3900316 (Tokyo)
	EPI_ISL_4204297 (2021-08-31)	ORF8:A65V	EPI_ISL_4646073 (Hyogo)
Sweden	EPI_ISL_4535910 (2021-09-16, Stockholm), EPI_ISL_4867614 (2021-09-16, Vasternorrland)	ORF8:P93S, Spike:A846G, Spike:Q677H	EPI_ISL_3899038 (Tokyo)
France	EPI_ISL_4283365 (2021-08-16, Provence-Alpes-Cote d’Azur)	ORF8:P93S	EPI_ISL_2723565 (Tokyo)
Australia	EPI_ISL_5033185 (2021-08-25, New South Wales)	ORF8:P93S, M:E167K, NSP16:A34V, NSP3:T350I, NSP4:K12R	EPI_ISL_4649671 (Kumamoto)
Belgium	EPI_ISL_3800062 (2021-08-23, Limburg), EPI_ISL_4031107 (2021-08-31, Limburg)	ORF8:P93S, NSP4:S481L, NSP8:A18T	EPI_ISL_4759372 (Hyogo)
Hong Kong	EPI_ISL_3219439 (2021-07-18)	ORF8:P93S	EPI_ISL_2723565 (Tokyo)
	EPI_ISL_3547114 (2021-08-14)	ORF8:P93S	EPI_ISL_2723565 (Tokyo)
Indonesia	EPI_ISL_5022763 (2021-09-11, West Java)	ORF8:P93S, NSP12:T26I, NSP4:T60I, NSP8:D101A	EPI_ISL_4641635 (Gifu)
Italy	EPI_ISL_3399092 (2021-08-14)	NSP3:P402T, NSP16:H174R, NSP7:A80V	EPI_ISL_3882160 (Tokyo)
New Zealand	EPI_ISL_3506222 (2021-08-10, Auckland), EPI_ISL_3506223 (2021-08-10, Auckland), EPI_ISL_3543461 (2021-08-17, Counties Manukau), EPI_ISL_3664424 (2021-08-19, Auckland), EPI_ISL_3543458 (2021-08-17, Auckland), EPI_ISL_3709130 (2021-08-21, Counties Manukau)	ORF8:P93S, Spike:T719I	EPI_ISL_2723565 (Tokyo)
Peru	EPI_ISL_4417371 (2021-08-19, Lima)		EPI_ISL_2768526 (Kanagawa)
Spain	EPI_ISL_4951307 (2021-09-17, Madrid)	ORF8:P93S, Spike:R158G	EPI_ISL_2723565 (Tokyo)
Thailand	EPI_ISL_5655524 (2021-08-22)		EPI_ISL_2768526 (Kanagawa)
	EPI_ISL_6695529 (2021-09-27), EPI_ISL_6695527 (2021-09-29)	E:V62F, ORF3:L140F, ORF7a:P34S, NSP14:D345Y, NSP1:E148G	EPI_ISL_3882581 (Tokyo)
Philippines	EPI_ISL_5543733 (2021-07-28)		EPI_ISL_2768526 (Kanagawa)
	EPI_ISL_5545908 (2021-08-07), EPI_ISL_5557098 (2021-08-10)	ORF8:P93S, NSP7:T45I, Spike:T778A	EPI_ISL_4666215 (Tokyo)
Israel	EPI_ISL_5620348 (2021-08-07)	ORF8:P93S, ORF8:A65T	EPI_ISL_2723565 (Tokyo)
Turkey	EPI_ISL_5331236 (2021-09-28)	ORF8:P93S, NSP12:G108V, NSP3:N1587D, NSP3:T199I	EPI_ISL_2723565 (Tokyo)
Canary islands	EPI_ISL_6470526 (2021-09-09), EPI_ISL_6470525 (2021-09-13), EPI_ISL_6470512 (2021-09-15)	Spike:S1097L	EPI_ISL_4722362 (Tokyo)

*118 samples were found in 20 countries. For each individual introduction represented by a row, additional characteristic mutations and ancestral strain in Japan are shown.*

## Discussion

Although AY.29 strains have been identified outside of Japan, with limited knowledge, it is not certain how many of them were associated with Olympic and Paralympic cross-border travelers. Olympic and Paralympic-related travelers account for approximately one-third of the entire outbound travelers from Japan as implied in Supplementary Figure 2; therefore, the events have likely contributed to the cross-border transmissions to some degree. For instance, three AY.29 samples were found in the Spanish territory the Canary Islands, which is rather an unusual location with low traffic from Japan. Members of the Spanish Olympic swim team participated in the domestic swim meet right after the Tokyo Games (Keith, 2021). Furthermore, the 900 samples released from the Japanese National Institute of Infectious Diseases missing prefectural information, such as Tokyo, contain 61 distinct novel exogenous strains as shown in Supplementary Table 1, which is a considerably high rate. The ancestor of EPI_ISL_3471119 collected in Scotland belongs to such samples without prefecture assignments as shown in Table 1. Further information regarding these samples is necessary for an impartial scientific evaluation; therefore, more detailed information about the samples in question should be released by the submitter.

The number of positive cases in Japan made a significant decrease from the peak in August as the vaccination rate in Japan increased. Although many breakthrough cases were reported due to the Delta variant (Brown et al., 2021; Herlihy et al., 2021) and various reports that indicate compromised vaccine effectiveness against the Delta variant were released (Fowlkes et al., 2021; Mlcochova et al., 2021; Planas et al., 2021), it is incontrovertible that vaccines are quite effective against AY.29 strain. Therefore, leakages of AY.29 to vaccinated countries would not induce serious issues. Unfortunately, this might not be the case for low-income countries, where only a few percent of citizens are vaccinated against SARS-CoV-2 at the time of the events (Coronavirus Pandemic (COVID-19), 2020; Mathieu et al., 2021). AY.29 is highly infective and virulent to unvaccinated people as demonstrated in Japan, where 0.42% of case fatality rate was observed for the AY.29 dominant fifth wave (Coronavirus Pandemic (COVID-19), 2020). On the other hand, the fatality rate for the Alpha variant dominant fourth wave in Japan was 1.8% (Coronavirus Pandemic (COVID-19), 2020), but the reduced fatality rate attributes to vaccination rather than mutations in the Delta variant AY.29 (Scobie et al., 2021). Due to the diverse nature of the summer Olympic Games, participants are from 205 countries (Wikipedia contributors, 2021), and it is worth noting that 57 participating countries have vaccination rates below 10%. Countries with low vaccination rates, whose SARS-CoV-2 genomes have not been updated are listed in Table 2. Many African nations are mentioned in the table. Besides, in Africa only one in seven positive cases were reported according to WHO (World Health Organization [WHO], 2021); therefore, it is challenging to detect any outbreaks in general. More rigorous departure screenings for participants from unvaccinated countries might have been necessary. Samplings of genomes in these countries are almost none or very low according to GISAID (2021). It is known that climate affects the fitness of SARS-CoV-2, and countries with low vaccination rates might not provide a favorable environment for the propagations of AY.29 substrains since their climates are substantially different from that of Japan (Islam et al., 2020). Even if they are sampled, it is common to take months before being shared. At this point, there is no sign of a significant surge due to the exported AY.29 strains; however, it is important to keep monitoring this potentially devastating strain for the time being.

**TABLE 2:** List of Countries with vaccination rates lower than 10% on the August 1st otherwise noted, whose SARS-CoV-2 genomes have not been updated since the Olympic and Paralympic Games.

Country or region	Number of athletes	GISAID genome	Last sampled date	Fully vaccinated per 100
Afghanistan	5	86	5/29/2021	1.08 (August 20)
Algeria	41	18	6/2/2021	1.62 (August 20)
Angola	20	108	7/19/2021	2.09 (August 3)
Armenia	17	82	3/18/2021	1.74
Benin	7	133	7/23/2021	0.17 (August 3)
Burkina Faso	7	116	5/7/2021	0.06 (August 31)
Burundi	6	63	7/28/2021	N/A
Cape Verde	6	0	N/A	3.86 (August 3)
Central African Republic	2	4	2021	N/A
Chad	3	9	2021	0.04 (July 29)
Comoros	3	6	1/10/2021	4.66 (August 3)
Cote d’Ivoire	28	20	2/24/2021	N/A
Djibouti	4	240	5/20/2021	1.89 (August 5)
Eritrea	13	0	N/A	N/A
Eswatini	4	52	7/26/2021	2.36 (August 2)
Ethiopia	38	117	8/2/2021	N/A
Federated States of Micronesia	3	0	N/A	N/A
Guinea	5	59	7/7/2021	2.52
Guinea-Bissau	4	8	2021	0.12 (August 9)
Haiti	6	72	8/7/2021	0 (August 2)
Honduras	27	89	7/31/2021	3.31 (August 3)
Iraq	4	140	7/28/2021	1.20 (July 11)
Jamaica	50	151	5/30/2021	4.45 (August 6)
Kiribati	3	0	N/A	N/A
Kyrgyzstan	17	0	N/A	1.77
Lesotho	2	18	1/18/2021	1.49 (August 25)
Liberia	3	77	7/10/2021	N/A
Libya	4	84	3/3/2021	0.75 (August 18)
Madagascar	6	248	4/26/2021	N/A
Malawi	5	41	7/5/2021	0.71
Marshall Islands	2	0	N/A	N/A
Mauritania	2	0	N/A	0.39 (August 3)
Mozambique	10	263	7/7/2021	1.00 (August 3)
Namibia	11	178	7/13/2021	1.91
Nicaragua	8	0		2.95 (August 17)
Niger	7	24	4/1/2021	0.13 (August 9)
Palau	3	0	N/A	N/A
Palestine	5	95	4/9/2021	N/A
Sao Tome and Principe	3	0	N/A	5.31 (August 3)
Senegal	9	224	6/1/2021	1.76 (August 3)
Sierra Leone	4	54	6/7/2021	0.41 (August 27)
Solomon Islands	3	5	3/26/2021	1.56 (August 2)
Somalia	2	18	4/6/2021	0.57 (August 21)
South Sudan	2	87	7/24/2021	0.04 (July 19)
Sudan	5	49	7/3/2021	0.41 (July 18)
Syria	6	0	N/A	0.05 (July 9)
Tajikistan	11	0	N/A	0.58
Tanzania	3	0	N/A	0.17 (August 8)
Togo	4	248	7/31/2021	1.81 (August 3)
Uganda	25	595	8/9/2021	0.51 (August 20)
Uzbekistan	64	81	7/23/2021	3.63 (August 2)
Vanuatu	3	2	3/26/2021	0.04 (July 27)
Venezuela	44	46	7/7/2021	3.83 (July 12)
Virgin Islands	4	11	5/30/2021	N/A
Yemen	5	0	N/A	0.04 (July 27)
Zimbabwe	5	112	7/25/2021	5.29 (August 2)

## Data Availability Statement

Publicly available datasets were analyzed in this study. Data used in this study is available from GISAID and NCBI repositories.

## Author Contributions

TK conceived the idea, performed the research, analyzed the data, and wrote the manuscript. RT analyzed the data and wrote the manuscript. KK and AS supported the data analysis. MK and SI supervised the study. All authors interpreted the data, reviewed the manuscript, made refinements, and approved the submitted version.

## Conflict of Interest

TK, RT, and MK were employees of IBM. The remaining authors declare that the research was conducted in the absence of any commercial or financial relationships that could be construed as a potential conflict of interest.

## Publisher’s Note

All claims expressed in this article are solely those of the authors and do not necessarily represent those of their affiliated organizations, or those of the publisher, the editors and the reviewers. Any product that may be evaluated in this article, or claim that may be made by its manufacturer, is not guaranteed or endorsed by the publisher.

## References

[B1] Abe T. Arita M. (2021). Genomic surveillance in Japan of Ay.29—a new sub-lineage of Sars-Cov-2 delta variant with C5239t and T5514c Mutations. *medRxiv* [Preprint]. 10.1101/2021.09.20.21263869

[B2] Brown C. M. Vostok J. Johnson H. Burns M. Gharpure R. Sami S. (2021). Outbreak of Sars-Cov-2 infections, including Covid-19 vaccine breakthrough infections, associated with large public gatherings - Barnstable County, Massachusetts. *MMWR Morb. Mortal Wkly. Rep.* 70 1059–1062. 10.15585/mmwr.mm7031e2 34351882 PMC8367314

[B3] Coronavirus Pandemic (COVID-19) (2020). *Research and data to make progress against the world’s largest problems.* Available online at: OurWorldInData.org (accessed February 18, 2022).

[B4] The Tokyo Organising Committee of the Olympic and Paralympic Games (2021). *Covid-19 Positive Case List*. Tokyo: The Tokyo Organising Committee of the Olympic and Paralympic Games.

[B5] Elbe S. Buckland-Merrett G. (2017). Data, disease and diplomacy: Gisaid’s innovative contribution to global health. *Glob. Chall.* 1 33–46. 10.1002/gch2.1018 31565258 PMC6607375

[B6] Fowlkes A. Gaglani M. Groover K. Thiese M. S. Tyner H. Ellingson K. (2021). Effectiveness of Covid-19 vaccines in preventing Sars-Cov-2 infection among frontline workers before and during B.1.617.2 (Delta) variant predominance - Eight U.S. locations. *MMWR Morb. Mortal Wkly. Rep.* 70 1167–1169. 10.15585/mmwr.mm7034e4 34437521 PMC8389394

[B7] GISAID (2021). *10,985,872 Human Sequences Collected and Shared via GISAID Since 10 January 2020.* Available online at: https://www.gisaid.org/index.php?id=208 (accessed October 24, 2021).

[B8] Hasegawa M. Kishino H. Yano T. (1985). Dating of the human-ape splitting by a molecular clock of mitochondrial DNA. *J. Mol. Evol.* 22 160–174. 10.1007/BF02101694 3934395

[B9] Herlihy R. Bamberg W. Burakoff A. Alden N. Severson R. Bush E. (2021). Rapid increase in circulation of the Sars-Cov-2 B.1.617.2 (Delta) variant – Mesa County, Colorado. *MMWR Morb. Mortal Wkly. Rep.* 70 1084–1087. 10.15585/mmwr.mm7032e2 34383734 PMC8360276

[B10] Islam M. R. Hoque M. N. Rahman M. S. Alam A. S. M. R. U. Akther M. Puspo J. A. (2020). Genome-wide analysis of Sars-Cov-2 virus strains circulating worldwide implicates heterogeneity. *Sci. Rep.* 10:14004. 10.1038/s41598-020-70812-6 32814791 PMC7438523

[B11] Katoh K. Misawa K. Kuma K. Miyata T. (2002). Mafft: a novel method for rapid multiple sequence alignment based on fast fourier transform. *Nucleic Acids Res.* 30 3059–3066. 10.1093/nar/gkf436 12136088 PMC135756

[B12] Keith B. (2021). *Hugo Gonzalez among Spanish Olympians Racing at Spanish Open This Week: Swim Swam.* Available online at: https://swimswam.com/hugo-gonzalez-among-spanish-olympians-racing-at-spanish-open-this-week/ (accessed November 8, 2021).

[B13] Koyama T. Platt D. Parida L. (2020). Variant analysis of SARS-CoV-2 genomes. *Bull. World Health Organ.* 98 495–504. 32742035 10.2471/BLT.20.253591 PMC7375210

[B14] Mathieu E. Ritchie H. Ortiz-Ospina E. Roser M. Hasell J. Appel C. (2021). A global database of Covid-19 vaccinations. *Nat. Hum. Behav.* 5 947–953. 10.1038/s41562-021-01122-8 33972767

[B15] Michael Rajah M. Hubert M. Bishop E. Saunders N. Robinot R. Grzelak L. (2021). Sars-Cov-2 alpha, beta and delta variants display enhanced spike-mediated syncytia formation. *EMBO J.* 40:e108944. 10.15252/embj.2021108944 34601723 PMC8646911

[B16] Mlcochova P. Kemp S. A. Dhar M. S. Papa G. Meng B. Ferreira I. A. T. M. (2021). Sars-Cov-2 B.1.617.2 delta variant replication and immune evasion. *Nature* 599 114–119. 10.1038/s41586-021-03944-y 34488225 PMC8566220

[B17] National Institute of Infectious Disease (2021). *Current Situation of Infection.* Rockville, MD: National Institute of Infectious Disease.

[B18] Needleman S. B. Wunsch C. D. A. (1970). General method applicable to the search for similarities in the amino acid Sequence of two proteins. *J. Mol. Biol.* 48 443–453. 10.1016/0022-2836(70)90057-4 5420325

[B19] NHK (2021a). *Tokyo Olympic and Paralympic Games Committee Reported Status of New Coronavirus (in Japanese): NHK.* Available online at: https://www3.nhk.or.jp/news/html/20210929/k10013281651000.html (accessed October 24, 2021).

[B20] NHK (2021b). *Total Number of Covid-19 Cases in Tokyo Olympic and Paralympic Games Were 863. (in Japanese): NHK.* Available online at: https://www3.nhk.or.jp/news/html/20210908/k10013249701000.html (accessed October 24, 2021).

[B21] Planas D. Veyer D. Baidaliuk A. Staropoli I. Guivel-Benhassine F. Rajah M. M. (2021). Reduced sensitivity of Sars-Cov-2 variant delta to antibody neutralization. *Nature* 596 276–280. 10.1038/s41586-021-03777-9 34237773

[B22] R Core Team (2021). *R: A Language and Environment for Statistical Computing.* Vienna: R Foundation for Statistical Computing.

[B23] Rambaut A. Holmes E. C. Hill V. O’Toole Á McCrone J. T. Ruis C. (2020). A dynamic nomenclature proposal for Sars-Cov-2 to assist genomic epidemiology. *bioRxiv* [Preprint]. 10.1101/2020.04.17.046086 PMC7610519 32669681

[B24] Sanderson T. Barrett J. C. (2021). Variation at spike position 142 in Sars-Cov-2 delta genomes is a technical artifact caused by dropout of a sequencing amplicon. *medRxiv* [Preprint]. 10.1101/2021.10.14.21264847 PMC9117943 35634532

[B25] Scobie H. M. Johnson A. G. Suthar A. B. Severson R. Alden N. B. Balter S. (2021). Monitoring incidence of Covid-19 cases, hospitalizations, and deaths, by vaccination status - 13 U.S. Jurisdictions. *MMWR Morb. Mortal Wkly. Rep.* 70 1284–1290. 10.15585/mmwr.mm7037e1 34529637 PMC8445374

[B26] Shu Y. McCauley J. (2017). Gisaid: global initiative on sharing all influenza data – From vision to reality. *Euro Surveill.* 22:30494. 10.2807/1560-7917.ES.2017.22.13.30494 28382917 PMC5388101

[B27] Tokumasu R. Weeraratne D. Snowdon J. Parida L. Kudo M. Koyama T. (2021). Introductions and evolutions of Sars-Cov-2 Strains in Japan. *medRxiv* [Preprint]. 10.1101/2021.02.26.21252555

[B28] Wells C. R. Townsend J. P. Pandey A. Moghadas S. M. Krieger G. Singer B. (2021). Optimal Covid-19 quarantine and testing strategies. *Nat. Commun.* 12:356. 10.1038/s41467-020-20742-8 33414470 PMC7788536

[B29] Wikipedia contributors (2021). *2020 Summer Olympics.* Available online at: https://en.wikipedia.org/w/index.php?title=2020_Summer_Olympics&oldid=1056489490 (accessed November 15, 2021).

[B30] World Health Organization [WHO] (2021). *Six in Seven Covid-19 Infections Go Undetected in Africa.* Available online at: https://www.afro.who.int/news/six-seven-covid-19-infections-go-undetected-africa (accessed October 24, 2021).

[B31] Yomiuri Shimbun (2021). *Olympic and Paralympic Games Related Visitors Were Reduced to 1/3 Excluding Athletes (in Japanese).* Available online at: https://www.yomiuri.co.jp/olympic/2020/20210618-OYT1T50254. (accessed October 24, 2021).

[B32] Yu G. (2020). Using ggtree to visualize data on tree-like structures. *Curr. Protoc. Bioinformatics* 69:e96. 10.1002/cpbi.96 32162851

