# Supplementary material for: Cross-Border Transmissions of the Delta Substrain AY.29 During Tokyo Olympic and Paralympic Games
Source: Front Microbiol. 2022 Aug 3;13:883849. doi: 10.3389/fmicb.2022.883849 (PMC9382352; doi:10.3389/fmicb.2022.883849)
Supplement: Supplementary file 2 [file Data_Sheet_1.docx]

**Supplementary Material**

**Cross-Border Transmissions of the Delta Substrain AY.29
During Tokyo Olympic and Paralympic Games**

Takahiko Koyama ^1*^, Reitaro Tokumasu^2^, Kotoe Katayama^3^, Ayumu Saito^4^, Michiharu Kudo^2^, Seiya Imoto^3,4^

1. IBM TJ Watson Research Center, Yorktown Heights, NY 10583, USA

2. IBM Research - Tokyo, Tokyo, Japan

3. Laboratory of Sequence Analysis, Human Genome Center, The Institute of Medical Science, The University of Tokyo

4. Division of Health Medical Intelligence, Human Genome Center, The Institute of Medical Science, The University of Tokyo

*Correspondence to Takahiko Koyama (email: tkoyama@us.ibm.com, telephone: +1-914-945-1910)

**Supplementary Methods**

**Statical collection and visualization.**

For statistical collection of COVID-19 patients in Japan, the data was extracted from Our World in Data.(1) For statistical collection of COVID-19 patients and COVID-19 summary in Tokyo 2020 Olympic and Paralympic Games, data was collected from Tokyo Positive Case List by The Tokyo Organizing Committee of the Olympic and Paralympic Games.(2) We used the data which is 8-Sep-21 version; Supplementary Figure 1, Supplementary Table 2 and 3. For statistical collection of inbound and outbound traveler number in Japan, data was extracted from e-Stat.(3) In the Number of positive case identified at quarantine, the data from COVID-19 Situation Report COVID-19 Situation Report on October 23, 2021 by Ministry of Health, Labor and Welfare, Tokyo, Japan.(4)

**References**

1. Coronavirus Pandemic (Covid-19) [Internet]. OurWorldInData.org (2020).

2. The Tokyo Organising Committee of the Olympic and Paralympic Games. Covid-19 Positive Case List. (2021) September 8. Report No.

3. Statistics of Immigration Control [Internet]. Immigration Services Agency of Japan (2021) [cited February 8, 2021]. Available from: <http://www.moj.go.jp/isa/policies/statistics/toukei_ichiran_nyukan.html>.

4. Ministry of Health Labor and Welfare. Covd-19 Situation Report on October 23, 2021 (2021) [cited 2021 November 10]. Available from: <https://www.mhlw.go.jp/stf/newpage_21832.html>.


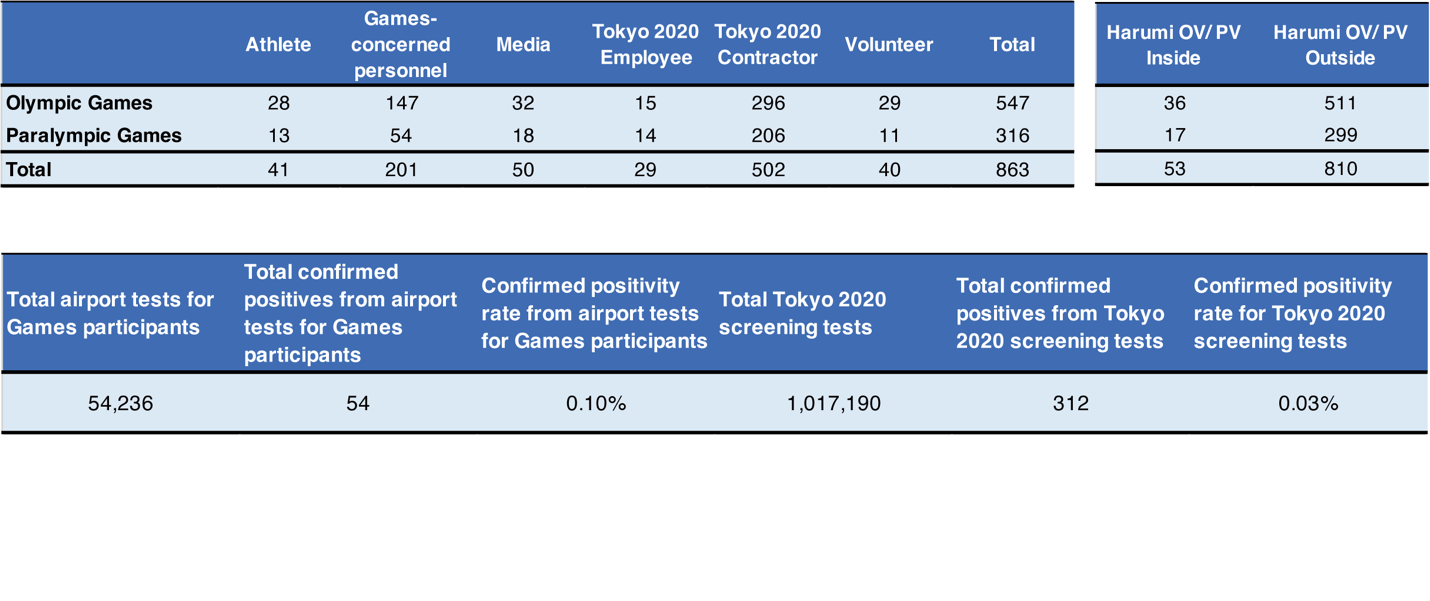


**Supplementary Figure 1. COVID-19 Summary in Tokyo 2020 Olympic and Paralympic Games.** Statistics of COVID-19 positive cases in the participants and COVID-19 tests performed for the participants


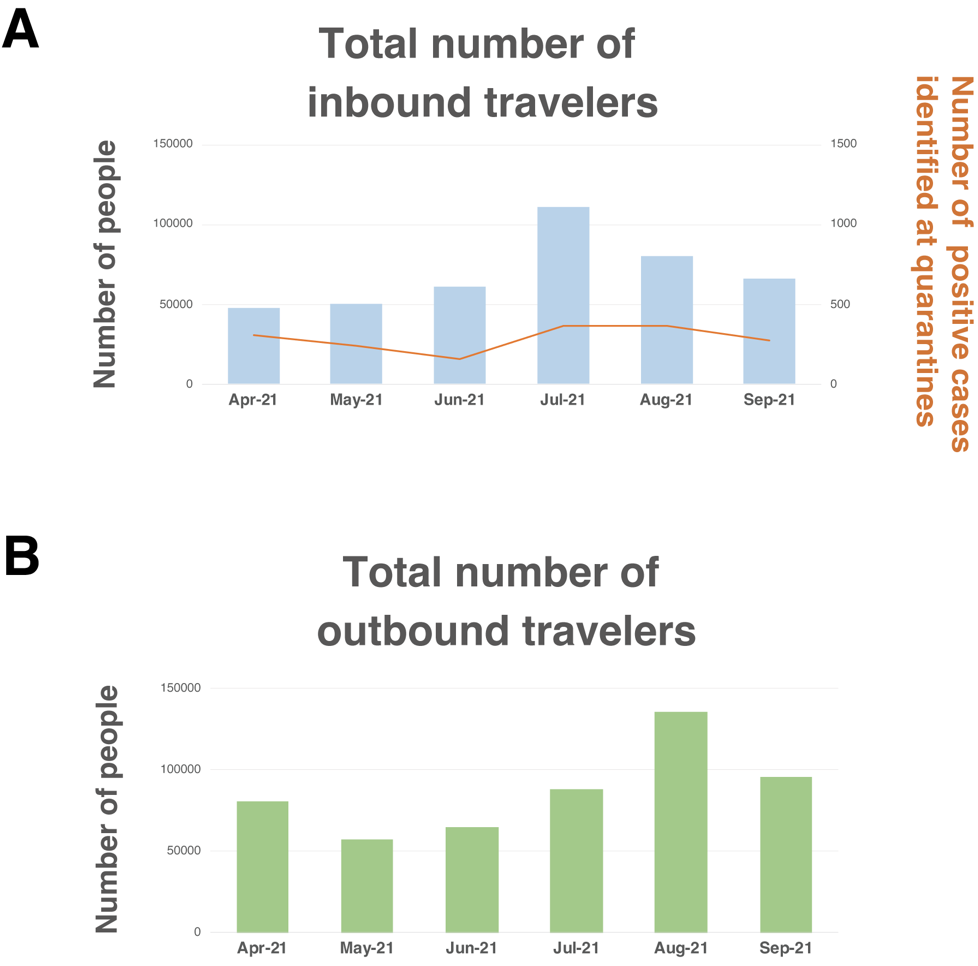


**Supplementary Figure 2. Statistical collection of inbound and outbound travelers in Japan.** A. Number of inbounds travelers to Japan between April and September 2021. B. Number of outbounds travelers from Japan between April and September 2021.

**Supplementary Table 1. Samples missing prefectural information during Olympic Games Tokyo 2020.**

| **Lineages** | **Number of Samples** | **Number of**  **Novel independent exogenous strains** |
| --- | --- | --- |
| AY.29 | 475 | 0 |
| B.1.1.7 | 63 | 0 |
| AY.98.1 | 37 | 1 |
| AY.29.1 | 35 | 0 |
| AY.122 | 34 | 7 |
| AY.68 | 30 | 1 |
| AY.60 | 27 | 2 |
| B.1.617.2 | 25 | 6 |
| AY.126 | 16 | 2 |
| AY.43 | 15 | 4 |
| AY.4.6 | 14 | 1 |
| AY.3.1 | 12 | 1 |
| AY.4 | 12 | 4 |
| AY.44 | 11 | 3 |
| AY.121 | 11 | 2 |
| AY.28 | 10 | 1 |
| AY.23 | 9 | 1 |
| AY.25 | 8 | 3 |
| P.1 | 8 | 1 |
| AY.47 | 6 | 1 |
| AY.20 | 5 | 1 |
| AY.45 | 5 | 1 |
| AY.109 | 5 | 1 |
| AY.34 | 4 | 1 |
| AY.122.1 | 4 | 1 |
| AY.119 | 3 | 2 |
| AY.46 | 3 | 2 |
| AY.1 | 3 | 1 |
| AY.33 | 2 | 2 |
| AY.42 | 1 | 1 |
| AY.34.1 | 1 | 1 |
| AY.36 | 1 | 1 |
| AY.37 | 1 | 1 |
| AY.74 | 1 | 1 |
| AY.75.3 | 1 | 1 |
| B.1.351.3 | 1 | 1 |
| AY.5 | 1 | 1 |

**Supplementary Table 2. Statistical collection of Covid-19 Positive Case List in Olympic Games Tokyo 2020.**

| **Date of announcement** | **Athlete** | **Games-concerned personnel** | **Media** | **Tokyo 2020 employee** | **Tokyo 2020 Contractor** | **Volunteer** | **Total** | **Harumi OV/ PV Inside** | **Harumi OV/ PV Outside** |
| --- | --- | --- | --- | --- | --- | --- | --- | --- | --- |
| 01-07-21 | 0 | 0 | 0 | 0 | 0 | 0 | 0 | 0 | 0 |
| 02-07-21 | 0 | 0 | 0 | 1 | 1 | 0 | 2 | 0 | 2 |
| 03-07-21 | 0 | 0 | 0 | 0 | 0 | 0 | 0 | 0 | 0 |
| 04-07-21 | 0 | 0 | 0 | 0 | 0 | 0 | 0 | 0 | 0 |
| 05-07-21 | 0 | 0 | 1 | 1 | 5 | 0 | 7 | 0 | 7 |
| 06-07-21 | 0 | 0 | 0 | 0 | 3 | 0 | 3 | 0 | 3 |
| 07-07-21 | 0 | 1 | 0 | 1 | 0 | 0 | 2 | 0 | 2 |
| 08-07-21 | 0 | 0 | 0 | 0 | 2 | 0 | 2 | 0 | 2 |
| 09-07-21 | 0 | 1 | 0 | 0 | 0 | 0 | 1 | 0 | 1 |
| 10-07-21 | 0 | 1 | 0 | 0 | 0 | 0 | 1 | 0 | 1 |
| 11-07-21 | 0 | 0 | 0 | 0 | 0 | 0 | 0 | 0 | 0 |
| 12-07-21 | 0 | 0 | 0 | 0 | 0 | 0 | 0 | 0 | 0 |
| 13-07-21 | 0 | 0 | 0 | 0 | 0 | 0 | 0 | 0 | 0 |
| 14-07-21 | 0 | 0 | 0 | 0 | 2 | 0 | 2 | 0 | 2 |
| 15-07-21 | 1 | 1 | 0 | 0 | 4 | 0 | 6 | 0 | 6 |
| 16-07-21 | 0 | 1 | 0 | 0 | 3 | 0 | 4 | 0 | 4 |
| 17-07-21 | 0 | 6 | 2 | 0 | 7 | 0 | 15 | 1 | 14 |
| 18-07-21 | 3 | 5 | 1 | 0 | 1 | 0 | 10 | 2 | 8 |
| 19-07-21 | 0 | 1 | 1 | 0 | 1 | 0 | 3 | 0 | 3 |
| 20-07-21 | 1 | 1 | 0 | 0 | 6 | 1 | 9 | 1 | 8 |
| 21-07-21 | 1 | 2 | 0 | 0 | 6 | 0 | 9 | 1 | 8 |
| 22-07-21 | 2 | 4 | 0 | 0 | 6 | 0 | 12 | 4 | 8 |
| 23-07-21 | 3 | 10 | 3 | 0 | 3 | 0 | 19 | 3 | 16 |
| 24-07-21 | 0 | 2 | 0 | 0 | 14 | 0 | 16 | 1 | 15 |
| 25-07-21 | 2 | 6 | 1 | 0 | 1 | 0 | 10 | 2 | 8 |
| 26-07-21 | 2 | 7 | 0 | 0 | 4 | 0 | 13 | 2 | 11 |
| 27-07-21 | 1 | 3 | 0 | 1 | 1 | 0 | 6 | 3 | 3 |
| 28-07-21 | 0 | 4 | 2 | 0 | 10 | 1 | 17 | 0 | 17 |
| 29-07-21 | 3 | 6 | 0 | 0 | 15 | 0 | 24 | 4 | 20 |
| 30-07-21 | 3 | 4 | 1 | 0 | 15 | 4 | 27 | 3 | 24 |
| 31-07-21 | 0 | 7 | 0 | 0 | 14 | 0 | 21 | 0 | 21 |
| 01-08-21 | 0 | 5 | 0 | 0 | 11 | 1 | 17 | 0 | 17 |
| 02-08-21 | 0 | 6 | 1 | 0 | 9 | 1 | 17 | 0 | 17 |
| 03-08-21 | 1 | 5 | 1 | 0 | 10 | 1 | 18 | 1 | 17 |
| 04-08-21 | 4 | 2 | 0 | 0 | 19 | 4 | 29 | 3 | 26 |
| 05-08-21 | 1 | 7 | 3 | 2 | 16 | 2 | 31 | 1 | 30 |
| 06-08-21 | 0 | 6 | 1 | 1 | 19 | 2 | 29 | 0 | 29 |
| 07-08-21 | 0 | 4 | 2 | 2 | 13 | 1 | 22 | 1 | 21 |
| 08-08-21 | 0 | 1 | 5 | 1 | 16 | 3 | 26 | 0 | 26 |
| 09-08-21 | 0 | 6 | 1 | 2 | 13 | 6 | 28 | 1 | 27 |
| 10-08-21 | 0 | 14 | 4 | 0 | 8 | 0 | 26 | 0 | 26 |
| 11-08-21 | 0 | 7 | 1 | 2 | 14 | 1 | 25 | 2 | 23 |
| 12-08-21 | 0 | 5 | 1 | 1 | 15 | 0 | 22 | 0 | 22 |
| 13-08-21 | 0 | 1 | 0 | 0 | 6 | 0 | 7 | 0 | 7 |
| 14-08-21 | 0 | 0 | 0 | 0 | 0 | 0 | 0 | 0 | 0 |
| 15-08-21 | 0 | 2 | 0 | 0 | 0 | 0 | 2 | 0 | 2 |
| 16-08-21 | 0 | 0 | 0 | 0 | 0 | 0 | 0 | 0 | 0 |
| 17-08-21 | 0 | 0 | 0 | 0 | 0 | 0 | 0 | 0 | 0 |
| 18-08-21 | 0 | 1 | 0 | 0 | 2 | 1 | 4 | 0 | 4 |
| 19-08-21 | 0 | 1 | 0 | 0 | 1 | 0 | 2 | 0 | 2 |
| 20-08-21 | 0 | 0 | 0 | 0 | 0 | 0 | 0 | 0 | 0 |
| 21-08-21 | 0 | 1 | 0 | 0 | 0 | 0 | 1 | 0 | 1 |
| 22-08-21 | 0 | 0 | 0 | 0 | 0 | 0 | 0 | 0 | 0 |
| 23-08-21 | 0 | 0 | 0 | 0 | 0 | 0 | 0 | 0 | 0 |
| 24-08-21 | 0 | 0 | 0 | 0 | 0 | 0 | 0 | 0 | 0 |
| 25-08-21 | 0 | 0 | 0 | 0 | 0 | 0 | 0 | 0 | 0 |
| 26-08-21 | 0 | 0 | 0 | 0 | 0 | 0 | 0 | 0 | 0 |
| 27-08-21 | 0 | 0 | 0 | 0 | 0 | 0 | 0 | 0 | 0 |
| 28-08-21 | 0 | 0 | 0 | 0 | 0 | 0 | 0 | 0 | 0 |
| 29-08-21 | 0 | 0 | 0 | 0 | 0 | 0 | 0 | 0 | 0 |
| 30-08-21 | 0 | 0 | 0 | 0 | 0 | 0 | 0 | 0 | 0 |
| 31-08-21 | 0 | 0 | 0 | 0 | 0 | 0 | 0 | 0 | 0 |
| 01-09-21 | 0 | 0 | 0 | 0 | 0 | 0 | 0 | 0 | 0 |
| 02-09-21 | 0 | 0 | 0 | 0 | 0 | 0 | 0 | 0 | 0 |
| 03-09-21 | 0 | 0 | 0 | 0 | 0 | 0 | 0 | 0 | 0 |
| 04-09-21 | 0 | 0 | 0 | 0 | 0 | 0 | 0 | 0 | 0 |
| 05-09-21 | 0 | 0 | 0 | 0 | 0 | 0 | 0 | 0 | 0 |
| 06-09-21 | 0 | 0 | 0 | 0 | 0 | 0 | 0 | 0 | 0 |
| 07-09-21 | 0 | 0 | 0 | 0 | 0 | 0 | 0 | 0 | 0 |
| 08-09-21 | 0 | 0 | 0 | 0 | 0 | 0 | 0 | 0 | 0 |

**Supplementary Table 3. Statistical collection of Covid-19 Positive Case List in Paralympic Games Tokyo 2020**.

| **Date of announcement** | **Athlete** | **Games-concerned personnel** | **Media** | **Tokyo 2020 employee** | **Tokyo 2020 Contractor** | **Volunteer** | **Total** | **Harumi OV/ PV Inside** | **Harumi OV/ PV Outside** |
| --- | --- | --- | --- | --- | --- | --- | --- | --- | --- |
| 12-08-21 | 0 | 0 | 0 | 0 | 0 | 0 | 0 | 0 | 0 |
| 13-08-21 | 0 | 2 | 0 | 0 | 2 | 0 | 4 | 0 | 4 |
| 14-08-21 | 0 | 1 | 1 | 2 | 8 | 0 | 12 | 0 | 12 |
| 15-08-21 | 0 | 1 | 1 | 1 | 5 | 0 | 8 | 0 | 8 |
| 16-08-21 | 0 | 0 | 1 | 1 | 5 | 0 | 7 | 0 | 7 |
| 17-08-21 | 0 | 1 | 0 | 0 | 8 | 0 | 9 | 0 | 9 |
| 18-08-21 | 0 | 1 | 4 | 1 | 11 | 1 | 18 | 0 | 18 |
| 19-08-21 | 0 | 3 | 0 | 1 | 12 | 0 | 16 | 1 | 15 |
| 20-08-21 | 1 | 1 | 1 | 2 | 7 | 0 | 12 | 0 | 12 |
| 21-08-21 | 1 | 5 | 0 | 0 | 9 | 0 | 15 | 1 | 14 |
| 22-08-21 | 2 | 8 | 2 | 1 | 16 | 1 | 30 | 0 | 30 |
| 23-08-21 | 0 | 3 | 0 | 1 | 7 | 0 | 11 | 2 | 9 |
| 24-08-21 | 1 | 2 | 1 | 1 | 5 | 0 | 10 | 2 | 8 |
| 25-08-21 | 2 | 5 | 1 | 0 | 8 | 0 | 16 | 5 | 11 |
| 26-08-21 | 2 | 3 | 0 | 0 | 10 | 0 | 15 | 2 | 13 |
| 27-08-21 | 0 | 2 | 2 | 0 | 9 | 0 | 13 | 0 | 13 |
| 28-08-21 | 0 | 5 | 0 | 0 | 14 | 3 | 22 | 0 | 22 |
| 29-08-21 | 2 | 1 | 1 | 0 | 7 | 0 | 11 | 2 | 9 |
| 30-08-21 | 1 | 1 | 0 | 1 | 7 | 1 | 11 | 1 | 10 |
| 31-08-21 | 1 | 1 | 1 | 1 | 10 | 0 | 14 | 1 | 13 |
| 01-09-21 | 0 | 1 | 0 | 0 | 5 | 1 | 7 | 0 | 7 |
| 02-09-21 | 0 | 2 | 0 | 0 | 9 | 2 | 13 | 0 | 13 |
| 03-09-21 | 0 | 1 | 0 | 1 | 8 | 2 | 12 | 0 | 12 |
| 04-09-21 | 0 | 1 | 0 | 0 | 9 | 0 | 10 | 0 | 10 |
| 05-09-21 | 0 | 1 | 1 | 0 | 2 | 0 | 4 | 0 | 4 |
| 06-09-21 | 0 | 1 | 0 | 0 | 5 | 0 | 6 | 0 | 6 |
| 07-09-21 | 0 | 1 | 1 | 0 | 4 | 0 | 6 | 0 | 6 |
| 08-09-21 | 0 | 0 | 0 | 0 | 4 | 0 | 4 | 0 | 4 |

**Supplementary Table 4. GISAID Genomes Used in Analysis**
The list is separately uploaded as a csv file in the supplementary material page.
